# On the Use of Creosote in Diseases of the Mucous Membranes, and in Discharges of Blood

**Published:** 1853-07

**Authors:** 


*On the Use of Creosote in Diseases of the Mucous Membranes, and in Discharges of Blood.* By Dr. Arendt.—The author points out the different affections of mucous membranes, in which striking benefit ensued from the administration of creosote. In ophthalmia chronica varicosa, in conjunctivitis with ulcers of the cornea, in opacities of the cornea, he directed a solution to be dropped daily into the eye (one or two drops to the ounce of water). He quieted cardial pains with two drops in eau sucré. In fluxus cœliacus, the discharge disappeared after repeated injections, (twenty-five drops to two pints of water.) Leucorrhœa ceased within a week after injections twice or thrice a day, (two drops to one ounce of water.) A similar injection was used with effect in catarrh of the bladder. A secondary post-puerperal discharge from the uterus he cured with an injection, (two drops to five ounces of water, to be used every two hours.) A good result also ensued in two cases of metrorrhagia connected with placenta prævia. In bloody discharges from the uterus of non-pregnant women, and in cases where it did not depend on the period of child-bearing or its after consequences, the injection of a solution of creosote was beneficial, but not always practicable, on account of the oblique position of the uterus, the presence of polypi, etc. Sometimes the source of the bloody discharge is beyond reach, as when it comes from the ovaries or the Fallopian tubes. In external hemorrhages the author found the remedy extremely useful, (ten to twenty drops to the ounce of water.)—*Med. Ztg. Rupl.*, 42, 43. 1852.—*Ibid.*

